# Norwegian GPs' participation in multidisciplinary meetings: A register-based study from 2007

**DOI:** 10.1186/1472-6963-10-309

**Published:** 2010-11-15

**Authors:** Øystein Hetlevik, Sturla Gjesdal

**Affiliations:** 1 Department of Public Health and Primary Health Care, University of Bergen, Kalfarveien 31, N-5018 Bergen, Norway

## Abstract

**Background:**

An increasing number of patients with chronic disorders and a more complex health service demand greater interdisciplinary collaboration in Primary Health Care. The aim of this study was therefore to identify factors related to general practitioners (GPs), their list populations and practice municipalities associated with a high rate of GP participation in multidisciplinary meetings (MDMs).

**Methods:**

A national cross-sectional register-based study of Norwegian general practice was conducted, including data on all GPs in the Regular GP Scheme in 2007 (N = 3179). GPs were grouped into quartiles based on the annual number of MDMs per patient on their list, and the groups were compared using one-way analysis of variance. Binary logistic regression was used to analyse associations between high rates of participation and characteristics of the GP, their list population and practice municipality.

**Results:**

On average, GPs attended 30 MDMs per year. The majority of the meetings concerned patients in the age groups 20-59 years. Psychological disorders were the motivation for 53% of the meetings. In a multivariate logistic regression model, the following characteristics predicted a high rate of MDM attendance: younger age of the GP, with an OR of 1.6 (95% CI 1.2-2.1) for GPs < 45 years, a short patient list, with an OR of 4.9 (3.2-7.5) for list sizes below 800 compared to lists ≥ 1600, higher proportion of psychological diagnosis in consultations (OR3.4 (2.6-4.4)) and a high MDM proportion with elderly patients (OR 4.1 (3.3-5.4)). Practising in municipalities with less than 10,000 inhabitants (OR 3.7 (2.8-4.9)) and a high proportion of disability pensioners (OR 1.6 (1.2-2.2)) or patients receiving social assistance (OR 2.2 (1.7-2.8)) also predicted high rates of meetings.

**Conclusions:**

Psychological problems including substance addiction gave grounds for the majority of MDMs. GPs with a high proportion of consultations with such problems also participated more frequently in MDMs. List size was negatively associated with the rate of MDMs, while a more disadvantaged list population was positively associated. Working in smaller organisational units seemed to facilitate cooperation between different professionals. There may be a generation shift towards more frequent participation in interdisciplinary work among younger GPs.

## Background

Chronic diseases represent 77% of the disease burden in Europe[1]. Care of patients with longstanding illness is therefore one of the main responsibilities of general practitioners (GPs). Health services have become more complex involving numerous professional groups, which demands more collaborative health and social services [2-5].

Studies have indicated that well-functioning multidisciplinary teamwork gives positive health outcomes for some patient groups [4-8]. Several barriers to the development of multidisciplinary collaboration have been identified, and the structure of the team, clearly defined roles and knowledge of each other's responsibilities are crucial for the team to function effectively [9,10]. A GP's participation in a multidisciplinary team attending one of his/her patients is regarded as important in providing an appropriate, coordinated service [11]. However, incorporating physicians in multidisciplinary work seems to be a special challenge [12,13].

With the introduction of a national regular GP scheme in Norway in 2001 all inhabitants were given the right to have a GP as their regular doctor [14]. A main aim of the reform was to improve health services for patients with chronic illnesses. In 2007, 3891 GPs contracted to municipalities had list sizes of 500 to 2500 patients. Norwegian GPs are mostly self-employed with 85% working in group practices, organised independently of other local health services. In their regular practices, Norwegian GPs are mainly paid by fee for services provided, but also have a fixed payment per patient on the list, estimated to give one third of GPs' income. A GP can claim a fee from the National Insurance Services when participating in a meeting with other professionals within health or social services as a part of patient treatment for a specified patient. The authorities have gradually increased the reimbursement for attending multidisciplinary meetings (MDMs) to approximately €100 per hour at present, which is comparable to income per hour in regular practice.

Since 2001, Norwegian patients who requires long-term, coordinated health services are entitled to have an individual plan (IP) according to The Patients' Rights Act [15]. A recommended working method is to set up a multidisciplinary team comprising health and social workers involved in the care of the patient. The patient should also be an active participant in the team. In most cases the work with IP is organised by municipalities, as shown in table 1.

**Table 1:** Individual plan - "The Norwegian model" for cooperation in Primary health care

1. According to the Patients' Rights Act*s *municipal health services have the responsibility to set up individual plans (IPs) for "*patient who requires long-term, coordinated health service"*, http://www.ub.uio.no/ujur/ulovdata/lov-19990702-063-eng.pdf)
2. Municipalities have a coordinating unit responsible for handling initiatives from patients or health-professionals when an IP is wanted and starts the work with the IP for each patient.
3. A coordinator is appointed in agreement with the patient, normally a person already involved in the treatment or care. GPs are very seldom the coordinator, but are usually included in the process as a medical advisor.
4. A multidisciplinary team is established, uniquely composed for each IP, based on patient's needs and the services involved.
5. The coordinator summons the team one to four times a year to plan treatment, rehabilitation and care, and to clarify responsibilities and revise the IP when necessary.
6. In addition to the patient, and/or close relatives, the participants in the multidisciplinary teams are found among professions obligatory in every Norwegian municipality: Public health nurses Home service nurses Mental health workers Physiotherapists Occupational therapists General practitioners Social workers/children welfare workers Teachers or special teachers
7. In addition representatives from the specialist health care representatives from the National Insurance Office often participate

The work with IP are more fully described in documents from the Norwegian Directorate of Health, found at: http://www.helsedirektoratet.no/vp/multimedia/archive/00010/IS-1292_E_10745a.pdf

The Norwegian government are planning a reorganisation of the health services, "The Cooperation Reform", based on the assumption that "patients' needs for coordinated services are not being sufficiently met" [16] The health authorities are also concerned regarding GPs' involvement in multidisciplinary activities [16], based on evaluation reports of the health services, especially of the psychiatric services [17]. List size reduction is proposed as a main tool to improve GPs participation in multidisciplinary teams, without scientific evidence supporting this strategy [16].

The present study explored Norwegian GPs' participation in MDMs concerning their patients, and the health problems addressed. We wanted to investigate the widespread opinion that the frequency of GPs' participation in MDMs is generally low, and that the list size, an indicator of workload, explains possible differences.

The aim of the study was to identify the impact of characteristics of GPs, patient list populations and the practice municipalities on the frequency of the GPs' participation in MDMs.

## Methods

### Material

A national cross-sectional register-based study of Norwegian general practice was conducted. All Norwegian GPs participating in the Regular GP Scheme who had practised during the whole year 2007 (N = 3179) were included, with a total of 97,091 reported MDMs.

The data were obtained from the following three data sources:

From the *national GP register *we obtained information on the GP's age and gender, the practice municipality, information on list length and changes in list length, and the age and gender of the patients on the GP's list.

The national research database *"FD trygd" *contains data on income, education, and receipt of social security and social assistance benefits on all inhabitants. Based on the unique ID number for each person in the *national GP register*, Statistics Norway linked this information anonymously to all persons on the patient lists (N = 3,936,126).

The Norwegian *National Insurance Services *receives bills from GPs in the fee-for-service system. We obtained anonymised data on all bills from GPs concerning all patient-related activities including participation in MDMs. These bills include a GP identity, the patient age and gender, but no person identification. They also include information of the time used in the meetings and a diagnosis set by the GP according to the International Classification of Primary Health Care (ICPC) [18].

The study was approved by the Norwegian Data Inspectorate and the owners of the databases.

### Outcome variable

We estimated the annual rate of MDMs per patient on the GP's list. Based on this rate, GPs were divided into quartiles, and these groups were compared with respect to GP age and gender, list size, and sociodemographic characteristics of the patient list population. The annual rate of MDMs was dichotomised, defining the 25% of GPs with the highest annual rates as a "high rate group". This was used as the outcome variable in the logistic regression analyses.

### Explanatory variables

GPs were characterised by age and gender.

The lists were characterised by the number of patients and whether or not the list was open to new patients.

The list populations were characterised by the proportion of males, mean age in the list population, educational level, mean annual income, the proportion of disability pensioners, and the proportion of recipients of social assistance on the list. Educational level was measured by the proportion of patients in the list aged over 20 years with only basic education (≤ 9 years' education). Income level was measured as the mean annual income among those aged 20 and above.

The GP practice municipalities were grouped according to the number of inhabitants.

A GP practice profile was indicated by the proportion of all the GP's ordinary consultations that had a main diagnosis of a psychological problem according to the P-chapter in ICPC.

The MDM proportion with patients ≥ 67 years adjusted for differences in list population was estimated, and defined as "high" when the proportion of MDMs concerning a patient ≥ 67 years was equal to or higher than the proportion of list population ≥ 67 years.

### Statistics

One-way analysis of variance was used to compare the quartiles of GPs according to rates of MDMs.

Binary logistic regression was used to analyse associations between "high" rates of participation in multidisciplinary meetings and GP, list-population and municipality characteristics. First, univariate odds ratios were estimated for all variables; then three multivariate models were constructed by including gradually new sets of variables. There was no reason to assume a linear association between variables and outcome, so variables were categorised by dividing the GPs in three equal sized groups.

The statistical package STATA 11 was used for the analyses.

## Results

On average, GPs attended 30 MDMs a year. The majority of the meetings concerned patients in the age groups 20-59 years (Table 2), and the proportion of male and female patients was almost identical. When the patient was ≥ 70 years, 75% of meetings lasted less than 30 minutes, compared to 30% for patients < 70 years (not tabled).

**Table 2:** Distribution of multidisciplinary meetings attended by 3179 Norwegian GPs in 2007, according to diagnoses^1 ^and patient age

	Number of meetings (%) within different age groups					
	
**ICPC chapters: **^**1**^	All ages	0-19 years	20-39 years	40-59 years	60-79 years	≥80 years
A - General and unspecified	10187 (10.5)	1223 (11.2)	3663 (11.3)	3393 (10.6)	798 (7.3)	1101 (10.4)
K - Cardiovascular	4903 (5.1)	81 (0.7)	235 (0.7)	953 (3.0)	1393 (12.7)	2241 (21.1)
L - Musculoskeletal	11937 (12.3)	446 (4.1)	3048 (9.4)	5789 (18.1)	1528 (13.9)	1126 (10.6)
N - Neurological	5528 (5.7)	1098 (10.0)	1412 (4.4)	1696 (5.3)	936 (8.5)	386 (3.6)
P - Psychological	52113 (53.7)	6451 (58.6)	21835 (67.3)	17497 (54.6)	3771 (34.4)	2559 (24.1)
All other diagnoses	12423 (12.7)	1702 (15.4)	2248 (6.9)	2716 (8.4)	2550 (23.2)	3206 (30.2)

Sum	97091 (100)	11011 (100)	32441 (100)	32044 (100)	10976 (100)	10619 (100)

^1 ^Diagnosis based on The International Classification of Primary Care [ICPC]

Patients with psychological diagnoses were the focus of the majority of the meetings. In 12% of all meetings a diagnosis of drug or alcohol addiction was used, in 10% depression and in 10% a serious mental disorder.

**Figure 1:**
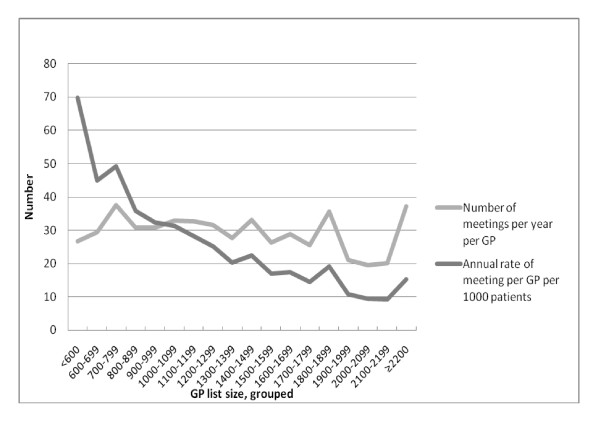
**Multidisciplinary meetings among 3179 Norwegian GPs**. *Mean annual number of meetings and rates per 1000 patients according to list size groups*.

Although the rates of MDMs clearly fell with larger list size (Figure 1), the mean annual number of meetings varied little between list size groups (min-max: 27-34).

Table 3 presents characteristics of the GPs, divided in quartiles according to rates of MDMs. The rate of meetings increased when the list had a higher proportion of list patients with low education, disability pensioners or social assistance receivers. There were only minor differences between groups in the distribution of diagnoses used in MDMs (not shown).

**Table 3:** GP, list, and list population characteristics among Norwegian GPs, grouped according to frequency of attendance in multidisciplinary meetings (MDMs) in 2007.

	**Frequency of attendance in MDMs**^**1**^				
	
	Low	Medium/low	Medium/high	High	p-value
Number of GPs	795	795	795	794	
Mean annual rate per 1000 patients (min-max)	4 (0-7)^2^	12 (7-17)	24 (17-35)	54 (35-170)	

***GP characteristics:***					
GP age, mean	52.2	50.0	48.5	47.3	< 0.001
GP gender: percent male	70.5	69.3	68.2	74.7	0.026

***GP practice characteristics:***					
Consultations with psychological diagnosis^3^,%	8.6	9.7	10.5	11.8	< 0.001
Rate MDMs, patients ≥67 years^4^	0.6	0.8	1.0	1.6	< 0.001

***List characteristics:***					
Mean list size	1367	1283	1225	1077	< 0.001
Percentage of lists open to new patients	32.7	28.6	26.9	37.0	< 0.001

***List population characteristics:***					
Persons ≥67 years on the list, %	12.9	12.3	12.1	13.0	0.014
Men in the list, %	49.4	49.2	49.2	51.1	< 0.001
Education ≤ 9 years^5^, %	14.1	14.4	14.6	15.9	< 0.001
Mean annual income (NOK1000)^5^	266	248	241	226	< 0.001
Disability pensioners^6^, %	9.5	10.1	10.7	11.7	< 0.001
Social assistance recipients^7^, %	3.6	4.1	4.6	5.6	< 0.001

One-way analyses of variance. N = 3179 GPs

1) GPs grouped in quartiles based on annual rate of multidisciplinary meetings per patient on the list

2) 83 GPs with no registered meetings

3) Proportion of all consultations in the GPs practices

4) Rate MDMs, patients ≥67 years = Proportion of MDMs concerning a patient ≥ 67 years/proportion of list population ≥ 67 years.

5) Proportion among patients ≥ 20 years

6) Proportion among patients 20-67 years

7) Proportion among patients 20-40 years

The univariate odds for being among the quartile of GPs with the highest rate of MDMs increased markedly with decreasing list size (Table 4). GPs with a list size of less than 800 patients had a 7.8 times higher unadjusted odds of being within the high rate group compared to GPs with a list size ≥ 1600 patients. Male GPs and younger age of the GP were also associated with a high rate of MDMs. There was an association between a high rate of meetings and lower socioeconomic level in the list population when measured by mean annual income or proportion of patients with a low educational level. The proportion of patients on disability pension or receiving social assistance benefits was also associated with high rates of MDMs. The rate of meetings was inversely associated with number of inhabitants in the practice municipality.

**Table 4:** List size and characteristics of GPs, list populations and practice municipalities associated with a high rate^1 ^of multidisciplinary meetings (MDMs) in Norwegian general practice.

		Univariate analyses			Model 1			Model 2			Model 3		
		
	*N*	*OR*	*95%CI*	*p-value*	*OR*	*95%CI*	*p-value*	*OR*	*95%CI*	*p-value*	*OR*	*95%CI*	*p-value*
***List size***													
≥ 1600	476	Ref			Ref			Ref			Ref		
1200 - 1599	1162	2.3	1.7-3.3	< 0.001	2.6	1.8-3.7	< 0.001	2.3	1.6-3.3	< 0.001	2.3	1.6-3.3	< 0.001
800 - 1199	1143	4.1	3.0-5.7	< 0.001	4.9	3.4-6.9	< 0.001	3.6	2.5-5.1	< 0.001	2.9	2.0-4.2	< 0.001
< 800	398	7.8	5.4-11.3	< 0.001	10.7	7.2-15.8	< 0.001	7.1	4.7-10.7	< 0.001	4.9	3.2-7.5	< 0.001

***GP age***													
≥ 55 years	1014	Ref			Ref			Ref			Ref		
45-54 years	1235	1.2	0.9-1.4	0.17	1.2	1.0-1.6	0.071	1.3	1.1-1.6	0.020	1.3	1.0-1.6	0.058
< 45 years	930	2.1	1.7-2.6	< 0.001	1.9	1.5-2.5	< 0.001	1.9	1.5-2.4	< 0.001	1.6	1.2-2.1	< 0.001

***GP gender***													
Female	932	Ref			Ref			Ref			Ref		
Male	2247	1.3	1.1-1.6	0.004	2.0	1.6-2.4	< 0.001	1.2	0.9-1.7	0.21	1.3	1.0-1.9	0.076

***MDM proportion with patients ≥ 67***													
Low ^2^	2225	Ref			Ref			Ref			Ref		
High ^3^	954	3.3	2.8-3.9	< 0.001	3.8	3.2-4.6	< 0.001	4.4	3.6-5.3	< 0.001	4.1	3.3-5.0	< 0.001

***Proportion of consultations*** with a psychological diagnosis*													
Low (< 8%)	1060	Ref			Ref			Ref			Ref		
Medium (8-11%)	1060	1.1	0.9-1.4	0.33	1.3	1.0-1.6	0.05	1.2	1.0-1.6	0.11	1.4	1.1-1.8	0.010
High (> 11%)	1059	2.4	2.0-2.9	< 0.001	2.8	2.2-3.4	< 0.001	2.7	2.1-3.4	< 0.001	3.4	2.6-4.4	< 0.001

Proportion ≥ 67 years*													
Low (< 9%)	1060	Ref						Ref			Ref		
Medium (9-15%)	1060	1.0	0.9-1.3	0.76				1.0	0.8-1.3	0.94	0.9	0.7-1.2	0.35
High (> 15%)	1059	1.2	1.0-1.5	0.056				1.0	0.8-1.4	0.80	0.9	0.6-1.2	0.39

Proportion male patients*													
Low (< 48%)	1060	Ref						Ref			Ref		
Medium (48-54%)	1060	1.5	1.2-1.9	< 0.001				1.6	1.2-2.3	0.002	1.3	1.0-1.9	0.076
High (> 54%)	1059	1.6	1.3-2.0	< 0.001				1.9	1.4-2.7	< 0.001	1.6	1.2-2.3	0.005

Proportion with education ≤ 9 years^4 *^													
Low (< 12%)	1060	Ref						Ref			Ref		
Medium (12-17%)	1060	1.0	0.9-1.3	0.71				0.7	0.5-0.8	0.001	0.6	0.4-0.8	< 0.001
High (> 17%)	1059	1.7	1.4-2.1	< 0.001				0.7	0.5-1.1	0.089	0.6	0.4-0.9	0.012

**Mean annual income among patients^4^**													
< 210,000 n.kr	799	Ref						Ref			Ref		
210000 - 250000	1283	0.7	0.6-0.8	< 0.001				0.8	0.6-1.0	0.046	0.9	0.7-1.2	0.40
> 250000	1097	0.3	0.3-0.4	< 0.001				0.5	0.3-0.6	< 0.001	0.7	0.5-0.9	0.008

Proportion disability pensioners^5 *^													
Low (< 8%)	1060	Ref						Ref			Ref		
Medium (8-11%)	1060	1.5	1.3-1.9	< 0.001				1.7	1.3-2.2	< 0.001	1.6	1.3-2.1	< 0.001
High (> 12%)	1059	1.9	1.6-2.4	< 0.001				1.7	1.2-2.6	0.001	1.6	1.2-2.2	0.005

Proportion with social assistance^6 *^													
Low (< 3%)	1060	Ref						Ref			Ref		
Medium (3-5%)	1060	1.4	1.1-1.8	0.002				1.1	0.9-1.4	0.37	1.2	0.9-1.6	0.14
High (> 5%)	1059	3.0	2.5-3.7	< 0.001				1.9	1.5-2.4	< 0.001	2.2	1.7-2.8	< 0.001

**Inhabitants in practice municipality**													
> 50,000	1103	Ref									Ref		
20-50,000	667	0.9	0.7-1.2	0.37							0.9	0.7-1.2	0.43
10-20,000	514	1.7	1.3-2.2	< 0.001							1.5	1.1-2.1	0.005
< 10,000	895	4.4	3.6-5.5	< 0.001							3.7	2.8-4.9	< 0.001

Binary logistic regression analyses. N = 3179 GPs

1) "High rate" defined as > 35 meetings per 1000 patients per year

2) Proportion of MDMs concerning patients ≥ 67 years lower than the proportion of list population ≥ 67 years

3) Proportion of MDMs concerning patients ≥ 67 years equal to or higher than the proportion of list population ≥ 67 years

4) Among list population ≥ 20 years

5) Proportion disability pensioners in the age group 20 - 67 years

6) Proportion with social assistance in the age group 20 - 40 years

*) Categorised variables with three equal sized groups of GPs, percentages are give, rounded to nearest whole numbers, to illustrate differences between GP groups.

In the first multivariate model, list size was adjusted for GP age and gender, and some practice characteristics, with an increase in the effect of list size.

In the second model, list size was also adjusted for list population variables, giving a markedly decrease in the list length effect. In this model GP gender showed no association with high rates of MDMs and the effect of several sociodemographic list population variables decreased or was eliminated.

When adjusting for all GP-, list-, population- and municipality characteristics, a smaller list size remained by far the strongest predictor of a high rate of MDM participation. However, a number of other variables were also shown to increase the odds for a high rate of MDMs: young GP age, a relatively high educational level in the list population, and a high proportion of disability pensioners and recipients of social assistance in the list. A higher proportion of ordinary GP consultations with a psychological diagnosis were associated with a high rate of MDMs. There was also a clear association between high MDM proportion concerning elderly and a high rate of meetings in general. Practising in municipalities with less than 10,000 inhabitants also predicted high rates of meetings when all other variables were adjusted for.

We also made a regression model including information of the GP being a specialist in family medicine or not, and length of practice time in the municipality, without revealing any significant associations with MDM rates (data not shown).

## Discussion

### Main findings

This register-based study on Norwegian GPs' participation in MDMs showed large differences between doctors. On average, GPs participated in one meeting every two weeks. Patients with a psychological diagnosis motivated the majority of the meetings.

The main predictors of a high rate of participation were a short or average list size and practising in a smaller municipality.

A high rate of MDMs was also associated with younger GPs, higher proportion of psychological diagnosis in ordinary GP consultations, higher MDM proportion concerning elderly patients and markers of lower socioeconomic status in the list population, except for education.

### The outcome variable, rates of multidisciplinary meetings

We used rates of participation in MDMs per patient on the list as the outcome measure. From a patient perspective, this is an indicator of the probability that the patient's GP will participate in multidisciplinary teams if necessary. Studies have indicated that GP participation in such collaboration is advantageous for patients [7]. Engaging physicians in multidisciplinary work has been shown to be a challenge, and there is a demand to increase GPs' participation based on possible health gain, perspectives of patient rights and political wishes [6,12,16,19,20]. One could argue that participating in multidisciplinary meetings is advantageous for patients with chronic diseases, based on findings in earlier studies [6-8,21,22]. Thus, the MDM rate is a measure of GP's adherence to "best practice" according to the Government's policy/guidelines for collaboration.

The rate of MDMs does not show the total amount of a GP's cooperation, which is also conducted by telephone or by written communication. However, meetings have been shown to be a facilitator of teamwork [10].

### GP variables

The study showed that younger GPs participated more frequently in MDMs. This could indicate a generation shift among Norwegian GPs, towards younger GPs working more closely with other professional groups, perhaps as a result of more emphasis on teamwork in the medical curriculum.

### The effect of list size

With the introduction of the regular GP Scheme, a list size of 1200-1500 patients was estimated to give a reasonable workload within the Norwegian PHC, were GPs are obliged to work one day a week in municipality health service for children or elderly. Workload is shown to increase with increasing list sizes [23,24]. GPs with larger list sizes have to prioritise more strongly in his or her work, and according to our findings, attendance in meetings may be given lower priority. It has been shown that it is necessary to allocate time to implement multidisciplinary teamwork [25], and that a high workload influences practice performance according to measures of quality of care [24,26].

On the other hand, teamwork may be a way of sharing responsibility for patient treatment. GPs have a central role in continuous care, as stated in the regulations of the GP scheme, and active cooperation with other health and social workers in patient treatment and care could ease GP workload and responsibility [12]. This perspective could motivate GPs to participate in team collaboration. However, this is not always included in the assessment when prioritising.

Although large list size seems to predict lower participation in MDMs, it is not obvious that reducing list sizes, as proposed by the Norwegian authorities, would change the GPs' practice. The ability to tackle a large list and the extent of collaboration may have a common explanation as a part of GPs' attitudes or practice styles shown to be GP-dependent in respect to other practice factors such as professional competence and time use [27]. However, this not possible to assess with register data

### Socioeconomic status

Low socioeconomic status in a population increases the prevalence of chronic diseases [28,29], and probably also the need for multidisciplinary coordinated services. When we adjusted for other variables in our model, the predictive value of low income among the list population was reduced, and low educational level became inversely associated with rate of MDMs. These variables are indicators of the disease burden in the total list population. Consequently list populations with a higher average socioeconomic level might have a lower disease burden, and the GPs may more easily engage in teamwork for the patients with special needs.

Being a disability pensioner or receiving social assistance implies a risk of serious health problems and also the need for an IP [30,31]. This study shows that the proportion of a list population dependent on state income supplements is a stronger predictor of a GP's participation in MDMs compared to socioeconomic variables aggregated at list population level.

### Psychological diagnoses

The motivation for most meetings was patients with psychological diagnoses. In this field, teamwork has been shown to be efficient in improving the outcome and quality of patient treatment [7,21,32]. Over the last decade, PHC has been given more responsibility in the treatment of patients with psychiatric diseases, and new groups of health workers are involved in both PHC and specialised care, resulting in more complex services. The high number of meetings within the field of mental health indicates that Norwegian GPs have responded to this challenge, and take an active role in teamwork concerning patients with mental health problems including drug and alcohol dependence. The study showed a strong association between a high proportion of ordinary consultations with psychological diagnoses and a high participation rate in MDMs. This can reveal differences in the mental health of the list population, but is probably also dependent on the GPs' field of interest.

### Elderly patients

The burden of disease is higher among elderly people, with an increasing need for coordinated health services, and a multidisciplinary approach has been shown to improve health outcome among the elderly [22]. The proportion of all the GPs' MDMs concerning elderly patients is relatively low, however, and does not reflect this need. IP is seldom used as a tool for cooperation among the elderly. Probably other channels, for instance within home nursing services and in nursing homes, are used in the collaboration for this patient group, without systematically involving the GPs.

A high proportion of elderly in the list had no impact on the general rate of participation in MDMs. Having a high MDM proportion with elderly patients was clearly associated with high rates of MDMs generally. Meetings concerning elderly patients were also generally shorter. These findings indicate local variation in cooperative routines for the elderly, where some GPs seems to be participating more frequently but in shorter meetings concerning elderly. Such local variations may explain a part of the large differences in MDM rates between GPs in general. Including more teamwork concerning elderly patients into practice routines is probably desirable and may contribute to higher rates of MDMs.

### Small municipalities

The association between high collaborative activity and practising in the smallest municipalities can be explained by the geographic and organisational nearness of services seen in these municipalities. These structural factors are not directly transferable to larger municipalities, but suggest that organising PHC based on a smaller geographical and organisational units, could improve multidisciplinary cooperation. Affiliating other health or social workers to GP group practices, as seen in the UK [33], may to some extent simulate a small municipality health service and could improve interdisciplinary cooperation.

### Strengths and Limitations

This study included all Norwegian GPs practising during the entire year 2007 and was based on register data for a full calendar year. This eliminates any selection bias of GPs' responses being influenced by his/her interest in collaboration or research in PHC.

The bills reporting meetings are reliable markers of this activity because the fee constitutes the sole payment and reporting is therefore probably complete. Over-reporting of meetings constitutes a criminal act, and is not very common.

Coding according to ICPC has been shown to be valid at chapter level and is assumed to reflect the actual health problems motivating the meetings [34,35].

A major limitation with our register data is the lack of information on the specific purpose of meetings and which other services were involved. We assume the majority of the meetings to be part of work with IP, in multidisciplinary teams, since this is the main organisational model for cooperation in PHC. However, GPs also participate in ad hoc meetings for patients in the need of coordinated services, when an IP is not established.

Because the GP bills were anonymised we did not know the number of different patients involved in the MDMs, but only the number of meetings, and age and gender of the patients involved.

Another weakness of the study is that no information was available on the number of patients with severe chronic disease in the list populations. We used sociodemographic indicators as a measure of health needs at list population level [29,36]. These aggregated indicators do not necessarily reflect diversities in the number of patients in need of multidisciplinary cooperation. The prevalence of mental health problems in the GPs' list populations was indicated by the proportion of ordinary GP consultations that was diagnosed with an ICPC diagnosis indicating psychological symptoms or disorders as the reason for the consultation.

We had no data on the GPs' skills or training in multidisciplinary work, attitudes to collaboration or the quality of the teamwork. Studies with a different design are necessary to explore such qualitative aspects. There was no information available about the organisation of practices, such as single-handed or group practice and the number of staff affiliated.

Finally, logistic regression analyses in cross-sectional samples reveal associations, and interpretation of causality must be made with caution.

### Further research

There is a need for more knowledge of the possible health effects of the Norwegian cooperation model based on IPs and multidisciplinary meetings. It is especially important to assess whether multidisciplinary team meetings are used for the patients with the highest needs for health service. The usefulness for patients of involving the GPs in MDMs should be studied, preferable in randomised controlled trials, which however seldom is feasible. Why younger GPs participate more in MDMs should also be studied, because this knowledge may be useful when designing educational programs for GPs in this area.

## Conclusion

Norwegian GPs have substantial engagement in multidisciplinary patient-centred meetings, with an average of one meeting every two weeks. However, rates of MDMs vary considerably between GPs. The present study identified several structural and GP related factors that influence GPs' multidisciplinary cooperation. These findings should be considered when policy changes for general practice are developed.

Positive health outcomes for patients with functioning multidisciplinary cooperation in their treatment and care have been documented. However, the benefit of teams seems dependent on structure and procedures [4,5,8,10,37,38].

## Competing interests

The authors declare that they have no competing interests.

## Authors' contributions

Both ØH and SG have contributed to the statistical analyses, to writing the manuscript and have approved the final version.

## Pre-publication history

The pre-publication history for this paper can be accessed here:

http://www.biomedcentral.com/1472-6963/10/309/prepub
